# Holistic description of new deep sea megafauna (Cephalopoda: Cirrata) using a minimally invasive approach

**DOI:** 10.1186/s12915-021-01000-9

**Published:** 2021-04-23

**Authors:** Alexander Ziegler, Christina Sagorny

**Affiliations:** Institut für Evolutionsbiologie und Ökologie, Rheinische Friedrich-Wilhelms-Universität, An der Immenburg 1, 53121 Bonn, Germany

**Keywords:** Magnetic resonance imaging, Micro-computed tomography, Three-dimensional, Modelling, Taxonomy, Cephalopod, Cirrate, Dumbo

## Abstract

**Background:**

In zoology, species descriptions conventionally rely on invasive morphological techniques, frequently leading to damage of the specimens and thus only a partial understanding of their structural complexity. More recently, non-destructive imaging techniques have successfully been used to describe smaller fauna, but this approach has so far not been applied to identify or describe larger animal species. Here, we present a combination of entirely non-invasive as well as minimally invasive methods that permit taxonomic descriptions of large zoological specimens in a more comprehensive manner.

**Results:**

Using the single available representative of an allegedly novel species of deep-sea cephalopod (Mollusca: Cephalopoda), digital photography, standardized external measurements, high-field magnetic resonance imaging, micro-computed tomography, and DNA barcoding were combined to gather all morphological and molecular characters relevant for a full species description. The results show that this specimen belongs to the cirrate octopod (Octopoda: Cirrata) genus *Grimpoteuthis *Robson, 1932. Based on the number of suckers, position of web nodules, cirrus length, presence of a radula, and various shell characters, the specimen is designated as the holotype of a new species of dumbo octopus, *G. imperator* sp. nov. The digital nature of the acquired data permits a seamless online deposition of raw as well as derived morphological and molecular datasets in publicly accessible repositories.

**Conclusions:**

Using high-resolution, non-invasive imaging systems intended for the analysis of larger biological objects, all external as well as internal morphological character states relevant for the identification of a new megafaunal species were obtained. Potentially harmful effects on this unique deep-sea cephalopod specimen were avoided by scanning the fixed animal without admixture of a contrast agent. Additional support for the taxonomic placement of the new dumbo octopus species was obtained through DNA barcoding, further underlining the importance of combining morphological and molecular datasets for a holistic description of zoological specimens.

## Background

Invasive methods to obtain morphological character states still form the cornerstone of metazoan species descriptions, despite the increased use of molecular techniques [1, 2]. For instance, describing a new cephalopod (Mollusca: Cephalopoda) species requires providing information on internal organs obtained through dissection [3]. Inevitably, this approach involves damage to or even partial destruction of the specimen at hand and therefore may preclude analysis of singular, endangered, rare, or otherwise valuable organisms. Furthermore, invasive techniques invariably alter the structural integrity of zoological specimens and thus do not permit studying organ systems in their natural context, ultimately resulting in a significantly restricted representation of the complexity of an organism. In contrast, digital three-dimensional (3D) imaging techniques such as computed tomography (CT) or magnetic resonance imaging (MRI) permit minimally or even entirely non-invasive analyses of whole biological specimens or parts thereof [4–8]. Although the application of such methods—an approach more recently labelled morphomics [9]—has so far been successfully demonstrated for the identification or description of smaller fauna [10–12], larger metazoan species have, for various technical or logistical reasons, thus far not been the target of this type of analysis.

Amongst cephalopods, the finned or dumbo octopuses (Octopoda: Cirrata) constitute relatively rare organisms, despite forming a significant part of the megafauna in deep-sea habitats of the World Ocean down to at least 7000 m depth [13]. With several cirrate species known only from a single collected specimen [14], a minimally invasive approach to describing new or redescribing existing taxa would constitute a significant improvement over conventional methods [15]. Based on the application of two complementary 3D imaging techniques supplemented by digital photography, standardized measurements of external features and DNA barcoding, we here present a novel workflow that results in the first minimally invasive, holistic description of a new metazoan species pertaining to a taxon composed entirely of megafaunal representatives.

## Methods

### Specimen collection, fixation, and preservation

The single cirrate specimen was collected in the North Pacific Ocean during scientific cruise SO-249 BERING (R/V SONNE) using a chain bag dredge [16]. The animal arrived dead on deck and was immediately transferred to a bucket filled with cold seawater. Several small tissue samples were collected from a single damaged arm using a scalpel and scissors. These tissue samples were then placed inside small plastic vials each filled with one of the following fixatives: 2 ml of 100% ethanol, 2 ml of 4% paraformaldehyde solution, 2 ml of 1:1 acetone/methanol solution, and 5 ml of RNAlater (Merck KGaA, Darmstadt, Germany). Following photography and measurements, the entire organism was placed inside a large plastic drum filled with 5 l of a 10% formalin solution prepared using distilled water supplemented with pH 7.2 microscopy buffer tablets (Merck KGaA). After several months, the specimen was transferred into 70% ethanol using a graded ethanol series. The specimen is deposited at the Museum für Naturkunde in Berlin, Germany (Zoologisches Museum Berlin Molluskensammlung, ZMB MOLL 240160).

### Video, photography, and standardized measurements

A short movie of the adult specimen arriving on deck (Additional file 1) was recorded using a Galaxy S7 smartphone with an integrated digital camera (Samsung Corp., Seoul, South Korea). The movie was edited using the Windows 10 Video-Editor software (Microsoft Corp., Redmond, CA, USA) and saved as an MPEG-4 file. In addition, the specimen was photographed on-board ship using a DSC-HX 400 digital camera with integrated flash (Sony Corp., Tokyo, Japan). Before fixation, external morphological characters were measured to the nearest millimetre using a ruler and calliper following established guidelines [3]. To document gross morphological changes potentially caused by fixation and preservation [14], the specimen was photographed prior to MRI after several months in the 10% formalin solution as well as prior to micro-computed tomography (µCT) after several months in the 70% ethanol solution.


**Additional file 1.** Movie file showing the extraction of the cirrate specimen from the chain bag dredge on-board R/V SONNE.

### Magnetic resonance imaging

For MRI, the formalin-fixed specimen was placed inside a cylindrical plastic container filled with the original 10% formalin solution. Scanning was performed using a 7-T high-field Magnetom clinical MR (magnetic resonance) system with a 600-mm magnet bore and equipped with an SC72 shielded gradient set with a maximum strength of 70 mT/m (Siemens, Berlin, Germany). The container was placed horizontally inside a 32-channel human head coil (Siemens). For imaging, a 3D magnetization-prepared spiral acquisition gradient echo (MP-SAGE) MR sequence with 3000 ms repetition time, 3.4 ms echo time, 7° flip angle, 37 frames averaged, 150 × 111 × 150 mm field of view, 536 × 396 × 536 px matrix size, 280 μm isotropic voxel resolution, and about 16 h 59 min acquisition time was used. The selected field of view comprised a region of interest extending from the posterior mantle edge to about the middle of the arms and thus covered all the internal structures relevant for a cirrate species description [3]. The acquired data were reconstructed using the software syngo MR B17 (Siemens). For further image processing, the original 16-bit NIfTI file was transformed into an 8-bit TIFF image stack, zero-filled to 140-μm isotropic voxel resolution, and finally rotated to a dorsoventral orientation using the software Fiji 1.52v [17].

### Micro-computed tomography

For μCT, the ethanol-preserved specimen was placed inside a cylindrical plastic container filled with the original 70% ethanol solution. Scanning was performed using a Phoenix v|tome|x s 180/240 CT system (GE Sensing & Inspection Technologies, Wunstorf, Germany) equipped with a 180-kV X-ray source and a detector measuring 2024 × 2024 px. Scan parameters were as follows: 100 kV source voltage, 100 μA source current, no filter, 500 ms exposure, 7 averages, 0.24° step size with 1500 projections over 360°, 13.2 × 14.4 × 13.2 mm field of view, 1210 × 1314 × 1204 px matrix size, 10.93 μm isotropic voxel resolution, and about 1 h 28 min acquisition time. The selected field of view focused on the buccal mass area. The resulting 16-bit projection images were reconstructed using the software Phoenix datos|x 2.7 (GE Sensing & Inspection Technologies). For further image processing, the original 16-bit RAW image volume was transformed into an 8-bit TIFF image stack and rotated to a sagittal orientation using the software Fiji 1.52v.

### Three-dimensional reconstruction and visualization

Based on the MRI 8-bit TIFF image stack, manual segmentation of selected internal organ systems was carried out using the *Segmentation Editor* in the software Amira 6.1 (Thermo Fisher Scientific Inc., Waltham, MA, USA). Surface rendering of the labelled organs was performed using the *SurfaceGen* module with *Constrained smoothing* activated. The resulting mesh was reduced from about 2,500,000 to 150,000 faces using the *Simplifier* function. Finally, the 3D model was saved as a WRL file using Amira’s *VRML-Export* module with *Render smooth* and *Render specular* activated. A similar approach was chosen to reconstruct the upper and lower beak based on the μCT 8-bit TIFF image stack. In addition, selected elements of the internal anatomy of the specimen were visualized in 3D using the *Volren* module in Amira. Virtual two-dimensional (2D) sections of the 8-bit MRI and μCT datasets were created using the Volume Viewer 2.0 plugin in the software Fiji 1.52v.

### Interactive 3D model creation

Two interactive 3D PDF files (Additional files 2 and 3) were created using the software Adobe 3D Reviewer 9.5.5 and Adobe Acrobat Pro Extended 9.5.5 (Adobe Systems Inc., San José, CA, USA). To this end, the WRL files exported from Amira were loaded into Adobe 3D Reviewer, where lighting, background, orientation, and labelling settings were adjusted. The models were each exported as PDF files, which were then loaded into Adobe Acrobat, where the cover image and pre-saved views were generated in order to finalize the interactive 3D PDF files. Please refer to previously published articles on how to use [18] or create interactive 3D PDF files [19–21].

### DNA extraction and sequencing

Genomic DNA was extracted from a single arm’s piece of tissue stored in 100% ethanol. DNA extraction was performed using the DNeasy Blood & Tissue Kit (Qiagen, Venlo, Netherlands) following the manufacturer’s extraction protocol. The mitochondrial 16S rRNA gene was amplified using the 16SarL (5′-CGCCTGTTTAACAAAAACAT-3′)/16SbrH (5′-CCGGTCTGAACTCAGATCACGT-3′) primer pair [22]. In addition, the mitochondrial cytochrome c oxidase subunit I (COI) gene was amplified using the LCO1490 (5′-GGTCAACAAAATCATAAAGATATTGG-3′)/HCO2198 (5′-TAAACTTCAGGGTGACCAAAAAATCA-3′) primer pair [23]. Polymerase chain reaction (PCR) was performed using Hot-Master Taq polymerase (Invitrogen, Carlsbad, CA, USA). PCR cycling was initiated with 2 min at 94 °C, followed by 35 cycles (40 s at 94 °C, 40 s at 50 °C, and 90 s at 72 °C), and terminated with a 2-min final elongation at 72 °C. Amplified products were purified using the NucleoSpin Gel and PCR Clean-up Kit (Macherey & Nagel, Düren, Germany) following the manufacturer’s instructions. Double-stranded Sanger sequencing [24] was conducted by a service provider (LGC Genomics, Berlin, Germany). Sequences were edited with BioEdit 7.2.5 [25] and aligned using MAFFT 7 [26]. For sequence alignment, the G-INS-I strategy with default parameters was chosen. All ambiguous positions were excluded with Gblocks 0.91b [27] using default parameters.

### Phylogenetic analysis

General time reversible (GTR) with gamma distribution was selected as the model for phylogenetic reconstruction using MrModeltest2 2.4 [28]. Phylogenetic trees were reconstructed in MEGA 6.06 using maximum likelihood (ML) as optimality criterion [29]. Branch support was estimated using 500 bootstrap replicates. Following an initial comparison, the 16S rRNA gene sequence was favoured over the COI gene sequence due to significantly broader taxon sampling in GenBank for the former and thus a better resolution of the resulting phylogeny. The 16S gene sequence of the new species (MW575539) was compared with one *Grimpoteuthis* sp. sequence (AF110100) from the North Pacific (note that this specimen is very likely an *Opisthoteuthis*), other *Grimpoteuthis* sp. sequences (AF487305-AF487312) from the North Atlantic (in fact all *G. discoveryi* – M. Collins, personal communication), and all further cirrate (Octopodiformes: Octopoda: Cirrata) 16S sequence data available from GenBank. In addition, further octopodiform specimens were incorporated as putative outgroup taxa, including several incirrate (Octopodiformes: Octopoda: Incirrata) and vampire squid (Octopodiformes: Vampyromorpha) specimens (Table 1).

**Table 1:** Octopodiform (Cephalopoda: Octopodiformes) taxa included in the phylogenetic analysis and their respective GenBank codes for the mitochondrial 16S rRNA gene sequence. New species marked in bold font

Family	Species	16S GenBank code
Cirroteuthidae Keferstein, 1866	*Cirroteuthis muelleri* Eschricht, 1836	AF487284
	*Cirrothauma murrayi* Chun, 1911	AF487282, AF487283
	*Stauroteuthis gilchristi* (Robson, 1924)	AF487291-AF487295, AY545102
	*Stauroteuthis syrtensis* Verrill, 1879	AF487285-AF487290, DQ280042
	*Stauroteuthis* sp.	AF487296
Grimpoteuthidae O’Shea, 1999	*Cryptoteuthis brevibracchiata* Collins, 2004	MT435502
	*Grimpoteuthis discoveryi* Collins, 2003	AF487305-AF487312
	***Grimpoteuthis imperator*** **sp. nov.**	**MW575539**
	*Grimpoteuthis* sp.	AF110100
	*Luteuthis dentatus* O’Shea, 1999	AJ315377
Cirroctopodidae Collins & Villanueva, 2006	*Cirroctopus glacialis* (Robson, 1930)	AF487304
	*Cirroctopus hochbergi* O’Shea, 1999	AJ315376
Opisthoteuthidae Verrill, 1896	*Opisthoteuthis californiana* Berry, 1949	AJ315373
	*Opisthoteuthis calypso* Villanueva et al., 2002	FJ403541, FJ403542
	*Opisthoteuthis chathamensis* O’Shea, 1999	MT216982
	*Opisthoteuthis depressa* Ijima & Ikeda, 1895	AB191117
	*Opisthoteuthis dongshaensis* Lu, 2010	AJ315375
	*Opisthoteuthis hardyi* Villanueva et al., 2002	AF487302, FJ785403, FJ785404
	*Opisthoteuthis massyae* (Grimpe, 1920)	AF487297-AF487301, AF299265, AJ315371, AJ315372, AY545103
	*Opisthoteuthis mero* O’Shea, 1999	MT216997, MT216998
	*Opisthoteuthis* sp.	AF487303, AF487304, AJ252768, AJ414702, AY616970
Bathypolypodidae Robson, 1929	*Bathypolypus arcticus* (Prosch, 1849)	DQ280044
Eledonidae Rochebrune, 1884	*Eledone moschata* (Lamarck, 1798)	AJ252764
Enteroctopodidae Strugnell et al., 2014	*Enteroctopus megalocyathus* (Gould, 1852)	HM572165
Megaleledonidae Taki, 1961	*Graneledone* sp.	JN800402
Octopodidae d’Orbigny, 1840	*Octopus bimaculatus* Verrill, 1883	KT335834
	*Octopus bocki* Adam, 1941	GQ900715
Vampyroteuthidae Thiele, 1915	*Vampyroteuthis infernalis* Chun, 1903	AY545101, AY686586, DQ280043, MG263918

## Results

The combined use of the non-invasive techniques digital photography, external measurements, MRI, and μCT complemented with DNA barcoding based on minimally invasive tissue sampling reveals that the single, well-preserved cirrate collected in the North Pacific Ocean is the first representative of a previously undescribed species of dumbo octopus. The full species description given below follows previously published guidelines [3] as well as two of the most recent descriptions of other new *Grimpoteuthis* species [30, 31]—please refer to the latter two articles for an explanation of the standardized abbreviations and the calculation of all relevant indices.

SYSTEMATICS

Family Grimpoteuthidae O’Shea, 1999

Genus *Grimpoteuthis* Robson, 1932

Type species: *Cirroteuthis umbellata* Fischer, 1883: 404. By original designation, Robson 1932: 137.

**Fig. 1:**
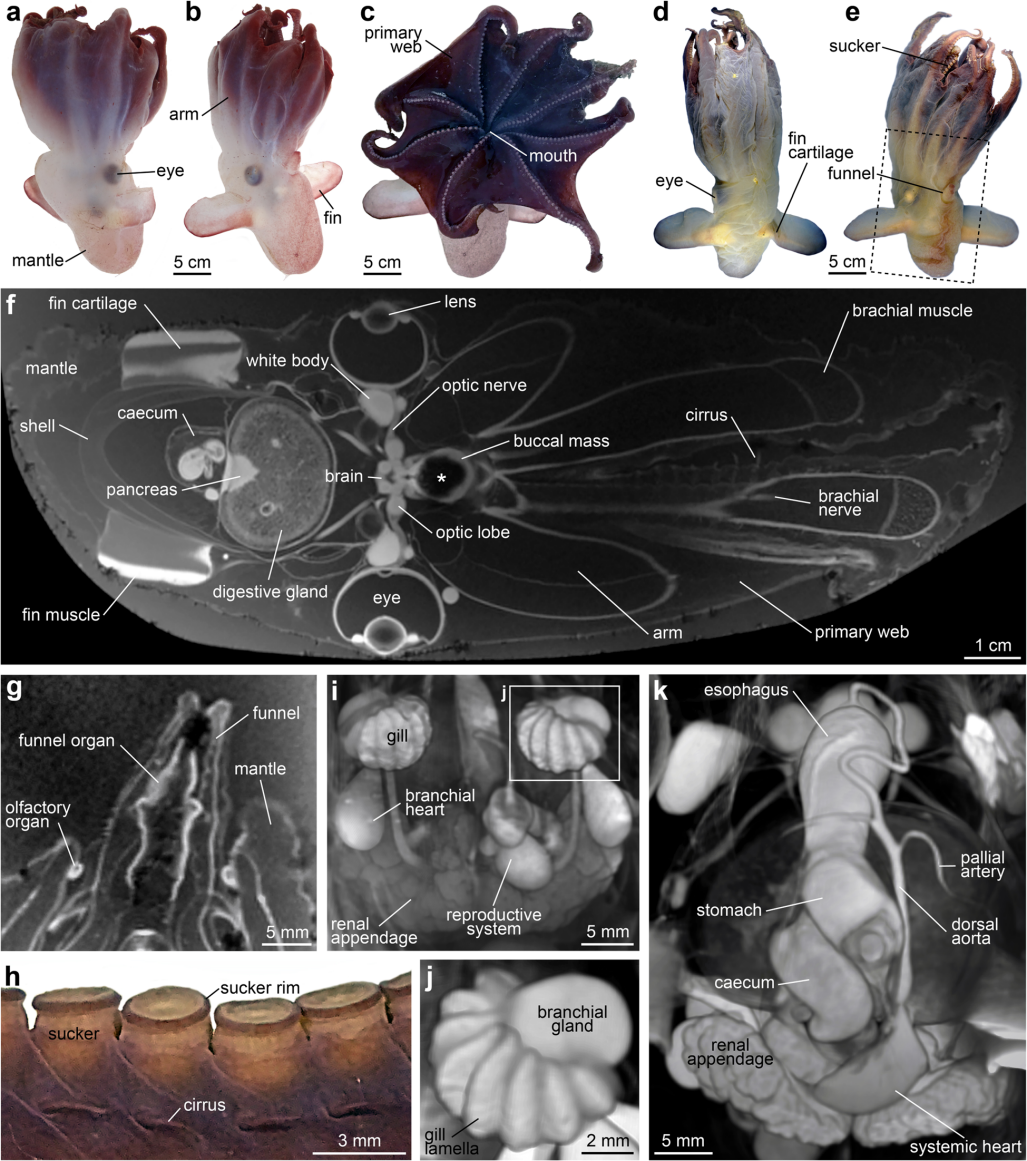
*Grimpoteuthis imperator* sp. nov. ZMB MOLL 240160. **a**–**c** Habitus before fixation showing dorsal, ventral, and oral views, anterior facing up. **d**, **e** Specimen prior to MRI following several months in 10% formalin solution showing dorsal and ventral views, anterior facing up. Stippled frame denotes the MRI region of interest. **f** Virtual section through the 3D MRI dataset, anterior facing right. The asterisk denotes a susceptibility artefact in the buccal mass area caused by ingested sediment. **g** Virtual section through the central long axis of the funnel. **h** Section of an arm showing the suckers and cirri, right lateral view. **i** Volume rendering of the viscera, ventral view, anterior facing up. **j** Close-up of the left gill showing eight broad lamellae. **k** Volume rendering of the viscera, oblique posterior view

**Fig. 2:**
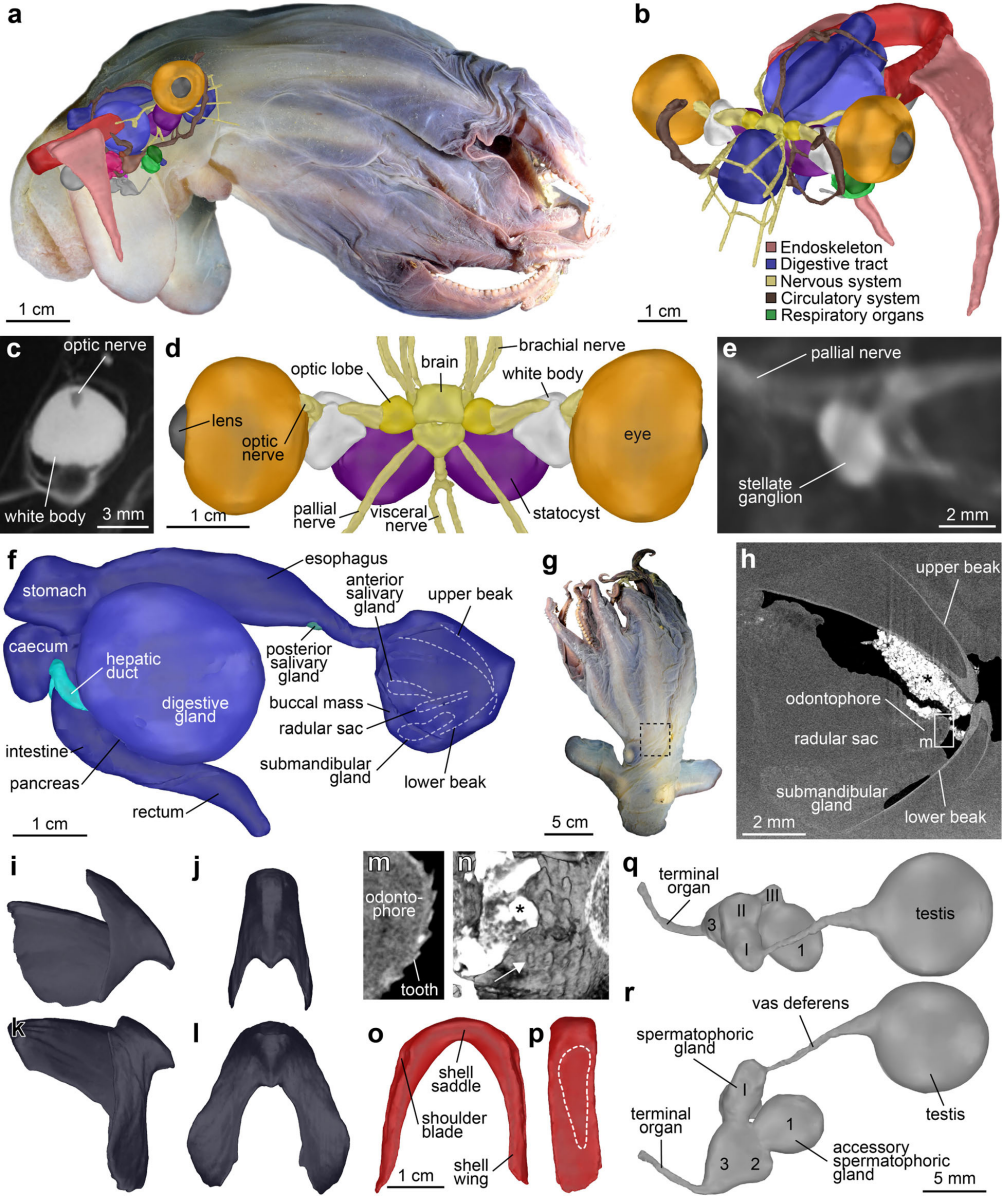
*Grimpoteuthis imperator* sp. nov. ZMB MOLL 240160. **a** Habitus prior to MRI following several months in 10% formalin solution showing a right lateral view, anterior facing right. The overlay of a lateral view of the surface-rendered 3D model (Additional file 2) illustrates relative size and position of the reconstructed organ systems. **b** Oblique anterior view of the entire 3D model of selected internal organs. **c** Virtual section through the left white body, anterior facing left. **d** Central nervous system and selected sensory organs, dorsal view, anterior facing up. **e** Volume rendering of the left stellate ganglion, anterior facing left. **f** Digestive tract with associated organs, right lateral view, anterior facing right. **g** Specimen prior to μCT following several months in 70% ethanol solution, dorsal view, anterior facing up. Stippled frame denotes the μCT region of interest. **h** Virtual section through the 3D μCT dataset, anterior facing right. The asterisk denotes ingested sediment. **i, j** Right lateral and oral views of the surface-rendered 3D model of the upper beak (Additional file 3). **k**, **l** Left lateral and oral views of the lower beak. **m** Virtual section through the 3D μCT dataset showing the anterior part of the radula in sagittal section. **n** Volume rendering of the radula, oral view. The asterisk denotes ingested sediment, arrow points to the rhachidian tooth. **o**, **p** Dorsal and right lateral views of the shell, anterior facing down. Stippled line denotes the fin cartilage insertion. **q, r** Dorsal and left lateral views of the male reproductive system, anterior facing left

*Grimpoteuthis imperator* sp. nov.

(Figs. 1 and 2; Tables 2 and 3; Additional files 1, 2, and 3; [32])

**Table 2:** Measurements and counts from the single male adult specimen of *Grimpoteuthis imperator* sp. nov. ZMB MOLL 240160. All measurements are provided in millimetres; values in parentheses represent measurements prior to fixation; asterisks denote damaged arm

	ZMB MOLL 240160
Total length (TL)	287 (290)
Mantle length (ML)	88 (95)
Mantle width (MW)	59 (61)
Head width (HW)	67 (69)
Fin span (FS)	195 (199)
Fin length (FL)	67 (69)
Fin width (FW)	34 (35)
Funnel length (FuL)	20 (21)
Eye diameter (ED)	17 (17)
Arm I length R/L	182/134*
Arm II length R/L	179/164
Arm III length R/L	156/166
Arm IV length R/L	157/157
Arm formula R/L (AF)	I.II.IV.III/III.II.IV.I*
Web depth sector A	95
Web depth sector B R/L	95/101
Web depth sector C R/L	87/92
Web depth sector D R/L	82/72
Web depth sector E	78
Web formula R/L (WF)	A = B.C.D.E/B.A.C.E.D
Web nodule location	33–34
Maximum sucker diameter (MSD)	3 (3)
Sucker count arm I R/L	73/38*
Sucker count arm II R/L	73/74
Sucker count arm III R/L	69/72
Sucker count arm IV R/L	70/68
Maximum cirrus length (MCL)	3 (3)
Location of proximal cirri between suckers	4–5
Gill diameter (GD)	8.8
Gill lamellae count R/L (GiLC)	8/8

**Table 3:** Comparison of *Grimpoteuthis imperator* sp. nov. with all other presently known species of *Grimpoteuthis*, listed in alphabetical order

Species	Type locality	*G. imperator* sp. nov differs in…
*G. abyssicola* O’Shea, 1999	Tasman Sea, South Pacific Ocean, 35° 35.1′ S, 160° 57.1′ E (https://tinyurl.com/uzuj4w9), 3145–3180 m depth	...having fewer suckers (68–74 vs. 77), shorter cirri (1.0× vs. 2.5× MSD), presence of a radula, and shell characters (weak shoulder blades vs. no shoulder blades, parallel shell wings vs. expanded shell wings)
*G. bathynectes* Voss & Pearcy, 1990	Tufts Abyssal Plain, North Pacific Ocean, 45° 01.1′ N, 135° 12.0′ W (https://tinyurl.com/w7b4nv2), 3932 m depth	...having more suckers (68–74 vs. 47–58), shorter cirri (1.0× vs. 1.1× MSD), presence of a radula, different position of web nodules (33–34 vs. 26), and shell characters (weak shoulder blades vs. no shoulder blades)
*G. boylei* Collins, 2003	Porcupine Abyssal Plain, North Atlantic Ocean, 48° 47′ N, 16° 30′ W (https://tinyurl.com/ubcxk5w), 4845–4847 m depth	...having more suckers (68–74 vs. 55–58), shorter cirri (1.0× vs. 1.9× MSD), different position of web nodules (33–34 vs. 31), and absence of enlarged suckers near web nodules
*G. challengeri* Collins, 2003	Porcupine Abyssal Plain, North Atlantic Ocean, 48° 56′ N, 15° 45′ W (https://tinyurl.com/u77dj8q), 4828–4838 m depth	...having shorter cirri (1.0× vs. 2.5× MSD), absence of enlarged suckers near web nodules, and shell characters (weak shoulder blades vs. well-developed shoulder blades)
*G. discoveryi* Collins, 2003	Porcupine Seabight, North Atlantic Ocean, 49° 35′ N, 14° 01′ W (https://tinyurl.com/tuz78ms), 4190–4255 m depth	...having more suckers (68–74 vs. 56–61), shorter cirri (1.0× vs. 1.2× MSD), presence of a radula, different position of web nodules (33–34 vs. 31), and absence of enlarged suckers near web nodules
*G. hippocrepium* Hoyle, 1904	Panama Basin, East Pacific Ocean, 02° 35′ N, 83° 53′ W (https://tinyurl.com/vl5rmjh), 3332 m depth	...having more suckers (68–74 vs. 50), different position of web nodules (33–34 vs. 25), and shell characters (weak shoulder blades vs. no shoulder blades, convex shell saddle vs. flat shell saddle)
*G. innominata* O’Shea, 1999	Chatham Rise, South Pacific Ocean, 42° 36.79′ S, 176° 09.81′ W (https://tinyurl.com/ux4stlu), 2000 m	...having more suckers (68–74 vs. 50–60), presence of a radula, different position of web nodules (33–34 vs. 22–24), gill shape (half-orange vs. semi-sepioid), and shell characters (weak shoulder blades vs. well-developed shoulder blades, parallel shell wings vs. expanded shell wings, convex shell saddle vs. convex shell saddle with median ridge)
*G. meangensis* Hoyle, 1885	Southwest off Pulau Kakalotan, South Pacific Ocean, 04° 33′ N, 127° 06′ E (https://tinyurl.com/rzfjdsm), 925 m depth	...having more suckers (68–74 vs. 60–70) and shell characters (weak shoulder blades vs. well-developed shoulder blades, convex shell saddle vs. flat shell saddle)
*G. megaptera* Verrill, 1885	Atlantic Abyssal Plain, North Atlantic Ocean, 36° 05.5′ N, 69° 51.8′ W (https://tinyurl.com/u8mnezr), 4600 m depth	...having shorter cirri (1.0× vs. 2.0× MSD)
*G. pacifica* (Hoyle, 1885)	Coral Sea, South Pacific Ocean, 13° 50′ S, 151° 49′ E (https://tinyurl.com/u9c8gx3), 4500 m depth	...having more suckers (68–74 vs. 52), shorter cirri (1.0× vs. 2.0× MSD), location of proximal cirri (between suckers 4–5 vs. 6–8), and absence of enlarged suckers near web nodules
*G. plena* Verrill, 1885	Atlantic Abyssal Plain, North Atlantic Ocean, 37° 35′ N, 71° 18.8′ W (https://tinyurl.com/vu3ynqs), 2000 m depth	...having more suckers (68–74 vs. 55), shorter cirri (1.0× vs. 1.2–1.6× MSD), and absence of enlarged suckers near web nodules
*G. tuftsi* Voss & Pearcy, 1990	Tufts Abyssal Plain, North Pacific Ocean, 45° 05.2′ N, 134° 43.4′ W (https://tinyurl.com/rf58bpj), 3900 m depth	...having shorter cirri (1.0× vs. 1.5–3.5× MSD), absence of enlarged suckers near web nodules, absence of a thin web between suckers, significantly shorter arms, and shell characters (weak shoulder blades vs. well-developed shoulder blades, convex shell saddle vs. convex shell saddle with transverse groove)
*G. umbellata* Fischer, 1883	Iberian Abyssal Plain, North Atlantic Ocean, 37° 55′ N, 20° 22′ W (https://tinyurl.com/rtxcfj8), 2235 m depth	...having more suckers (68–74 vs. 65–68) and shorter cirri (1.0× vs. 1.2× MSD)—note that the single (and LECTOTYPE) specimen is badly damaged and likely a *G. discoveryi* or *G. wuelkeri* [31]
*G. wuelkeri* Grimpe, 1920	Gulf of Cadiz, North Atlantic Ocean, 35° 46′ N, 08° 16′ W (https://tinyurl.com/ufb2cx8), 2055 m depth	...having shorter cirri (1.0× vs. 2.5× MSD), different position of the web nodules (33–34 vs. 28), gill lamellae count (8 vs. 6–7), and shell characters (weak shoulder blades vs. well-developed shoulder blades, convex shell saddle vs. convex shell saddle with median ridge)

### Synonymy

*Grimpoteuthis* sp. ([16]: 83, Fig. 114A)

*Grimpoteuthis* sp. ([33]: 265; Fig. 10F, G)

#### Material examined

HOLOTYPE: mature male, ML 95 mm; R/V SONNE, SO-249 BERING, DR 82; 48° 27.53′ N, 168° 52.21′ E, 3913–4417 m depth; chain bag dredge; 5 July 2016; ZMB MOLL 240160.

#### Diagnosis

Medium-sized species with moderately long, lateral fins. Cirri short and suckers moderate. Gills compact with eight broad lamellae. Radula present, teeth homodont. Paired anterior and unpaired posterior salivary glands present. Shell U-shaped, smooth with lateral wings parallel, broadly tapering towards distal ends.

#### Description

Medium-sized species, body semi-gelatinous, bell-shaped (Fig. 1a–e). Mantle about one third of total length (MLI 32.8), saccular, gelatinous, nearly twice as long as wide, broadly rounded posteriorly. Head wider than mantle (HWI 23.8), no neck region visible (Fig. 1f). Pallial aperture narrow, enveloping base of funnel (Fig. 1e). Funnel long (FuLI 30.4), protruding far beyond mantle margin, distal third of funnel free (Fig. 1b). Funnel broader at the base than at the opening (Fig. 1g). Funnel organ of inverted V shape. Olfactory organs rounded, situated within the pallial aperture on either side of the funnel base (Fig. 1g). Fins moderately long (FSI 68.6, FLI 100.0), about half as wide as long (Fig. 1b). Fins situated laterally, between the eyes and apex of the mantle, but closer to the eyes. Posterior fin margin straight, less gelatinous than the rest of the mantle. Tips of fins broadly rounded. Anterior fin margin slightly convex, tapering posteriorly towards the base. Fin cartilage and fin muscles inserting in the posterior portion of the fins (Fig. 1d). Broad muscular base, attached to the shell. Towards the tips of fins, the fin cartilage becomes narrower, ending in a small tip about ¾ of fin length.

Arms semi-gelatinous, subequal, moderately long (ALI 63.4). The fourth arm on ventral side shortest (ca. 54% of total length), first arm on dorsal side longest (ca. 63% of total length), arm formula varying between left and right sides. The arms deeply set into the soft and fleshy primary web, no secondary web present (Fig. 1c). Web formula differing between right and left sides with sector A or B deepest and sector D or E shallowest, respectively. Web nodules large and rounded, located on the ventral side of the arms between suckers 33 and 34, uniting web to the ventral side of the arms (Fig. 1c). Beyond the nodule, the web extends only a few suckers further, becoming very narrow. Nodules absent on the dorsal surface, web extending nearly to the tip of each arm. No nodules present in sector A, so the web extends nearly to the tip of both adjacent arms. In sector E, two nodules present, so the web extends only a short distance beyond the nodules, thus rendering this sector one of the shallowest sectors and sector A one of the deepest. Suckers in a single row extending from the mouth opening to the tips of the arms (Fig. 1c). Arms with 68–74 suckers set into the oral surface, their apertures projecting freely. First five suckers very small, followed by suckers gradually increasing in size (MSDI 4.3). No enlarged suckers around the web nodule, no sign of hectocotylization (Fig. 1c). Suckers cylindrical, with short, narrow apertural rims (Fig. 1h). Simple sucker aperture, the base of suckers embedded into primary web. Cirri short, 1.0× maximum sucker diameter (MCLI 4.3), located on the oral surface of the arms between base of suckers (Fig. 1h). First cirrus between suckers 4 and 5. Cirri extending to the tips of the arms with no change in length along the arms.

Gills compact (GDI 13.1), almost spherical (Fig. 1i), of ‘half-orange’ type, with eight broad lamellae each (Fig. 1j). Gills partly surrounding ovoid-shaped branchial glands. Teardrop-shaped branchial hearts located dorso-posterior to the gills, in close proximity to the renal appendages. Systemic heart with single large curved ventricle, indistinguishable auricles (Fig. 1k). Small swelling on the dorsal aorta directly dorsal to the ventricle. Dorsal aorta forming single large curve across the dorsal side of the oesophagus. Two large pallial arteries leaving the dorsal aorta as a single vessel each near the posterior end of the oesophagus. Eyes large (EDI 24.6), not protruding, with prominent lens (Fig. 2a, b). Optic lobes almost spherical in shape, with a single bundle of optic nerves passing through the white body (Fig. 2c) before tapering into several smaller nerves closer to the eye. White bodies more than twice the size of the optic lobes, somewhat spherical in shape with two processes directed towards the brain. Statocysts almost as large as the eyes (Fig. 2d). Stellate ganglion ovoid in shape, epistellar body morphologically not distinguishable (Fig. 2e).

The digestive tract in lateral view C-shaped with relatively large buccal mass (Fig. 2f–h). The upper beak without distinct folds, ridges, or thickenings (Fig. 2i, j). Lower beak with rounded hood and broad wings, lateral walls without any folds or ridges (Fig. 2k, l). Paired anterior salivary glands centrally located within the buccal mass, to the left and right side above the radular sac (Fig. 2f). Large median submandibular gland below radular sac (Fig. 2f, h). Radula poorly developed, with homodont dentition arranged in five rows (Fig. 2m, n). Rhachidian tooth slender, smaller than laterals. First lateral tooth broad triangular, second lateral tooth slender triangular, no trace of marginal teeth or plates. Oesophagus long, widening in the mid-part to form a simple crop (Fig. 2f). Unpaired posterior salivary gland small, located on the ventral side of the oesophagus. Stomach nearly rectangular, tapering towards the ventral side. The caecum slightly smaller than the stomach with two long and slender hepatic ducts leading to the large, unilobular, almost spherical digestive gland (Fig. 2f). Small pancreas present on the posterior surface of the digestive gland. Intestine shorter than the oesophagus, no enlargement. Rectum located close to the funnel. Anal flaps and ink sac absent.

Shell located in the dorsal part of the mantle cavity, robust, U-shaped (Fig. 2a, b). Shell wings not expanding, parallel to each other, broadly tapering towards the distal ends (Fig. 2o). Shoulder blades present, but weak. Outer surface of the shell saddle convex without median ridge or transverse groove. Fin cartilage insertions long, encompassing about 2/3 of the entire length of the shell wing (Fig. 2p).

Male reproductive system with large, almost spherical testis located centrally in the posterior part of the mantle, posterior to the stomach and caecum, ventral to the shell (Fig. 2a). Vas deferens elongated. Spermatophoric glands I–III moderately developed, convoluted (Fig. 2q). Accessory spermatophoric gland complex much larger than the spermatophoric gland complex. Accessory spermatophoric glands 1 and 3 nearly the same size, much larger than accessory spermatophoric gland 2 (Fig. 2r). Several spermatophores located inside the central duct within the accessory spermatophoric gland complex, each measuring ca. 2 × 1 mm. Terminal organ (penis) long and slender, directed towards the rectum (Fig. 2r).

Skin surface smooth. Dorsal surface of the head and mantle white, slightly reddish towards the posterior apex (Fig. 1a). Ventral surface of the head and mantle white with a red pigmentation, funnel darker red in colour, in particular at the distal end (Fig. 1b). Posterior margin of the fins deep red, becoming lighter and almost white towards the anterior margin. Dorsal arms on the surface white, dorsal surface of the web reddish (Fig. 1a). Ventral arms and web dark red, suckers lighter red (Fig. 1c).

#### Measurements and counts

Most morphometric and meristic characters were obtained from the holotype directly following capture (Table 2).

#### Type locality

On a southwest-facing slope above a large circular basin southeast of Tenji Seamount, east of Winnebago Seamount, and northeast of Minnetonka Seamount, Emperor Seamounts, North Pacific Ocean; 48° 27.53′ N, 168° 52.21′ E, 3913–4417 m depth (https://tinyurl.com/rmsp7ej).

#### Distribution

So far known only from the type locality in the northern part of the Emperor Seamounts, an undersea mountain chain in the northwestern part of the North Pacific [34].

#### Remarks

**Fig. 3:**
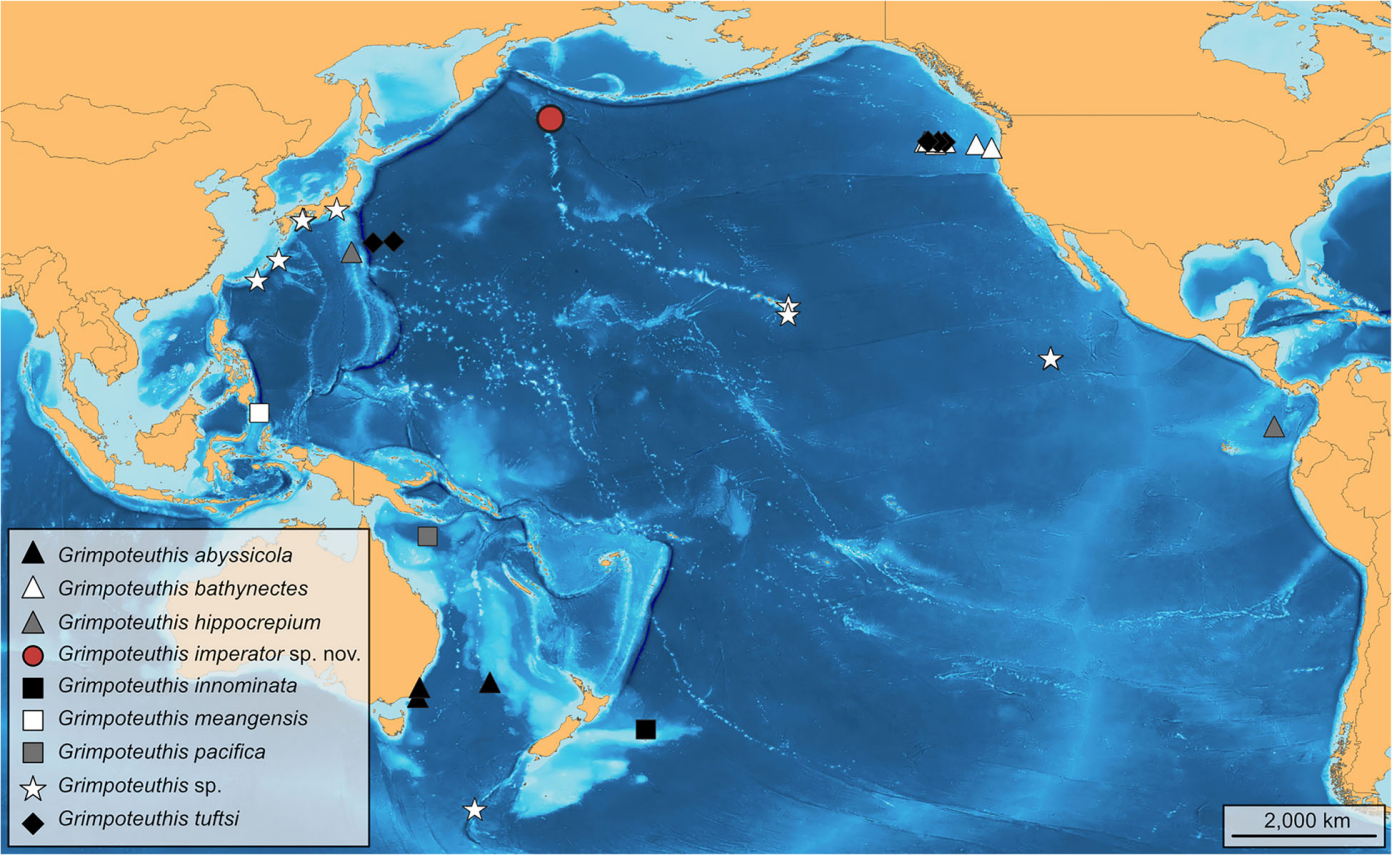
Geographic distribution of identified and unidentified specimens of *Grimpoteuthis* in the Pacific Ocean. See Table 3 for a list of the respective type localities

Based on the shell form, fin position, optic lobe shape, arm length, web form, and optic nerve arrangement, this specimen is readily identifiable as a cirrate of the genus *Grimpoteuthis* [14]. Differentiation from previously described species is based on the number of suckers (68–74), absence of enlarged suckers near the web nodules, absence of a thin web between the suckers, position of the web nodules (near suckers 33-34), cirrus length (1.0× maximum sucker diameter), location of proximal cirri (between suckers 4 and 5), presence of a radula, gill shape (‘half-orange’ type), gill lamellae count (8/8), and various shell characters (Table 3). Apart from the new species described here, seven other *Grimpoteuthis* species as well as several unidentified specimens ascribed to this genus have been recorded from the Pacific Ocean (Fig. 3). The type localities of the two geographically closest species, i.e. *G. bathynectes* and *G. tuftsi*, are also found in the North Pacific, but in areas more than 4000 km east of the type locality of *G. imperator* sp. nov. [30]. Further type localities of Pacific species pertaining to that genus are found in the tropical and southern parts of the ocean. Whilst only a single individual was analysed here, this is not uncommon in cirrate taxonomy due to the scarcity of suitable material [14] and under certain circumstances (e.g. ‘unquestioned distinctiveness of the species’ and ‘the high probability that no additional material will be forthcoming soon’) does not impede designation of a new species ([3]: 49).

#### Etymology

Latin, imperator, noun in apposition. Named after the Emperor Seamounts to which the type locality belongs. Proposed vernacular names are Emperor dumbo (English), Dumbo impérial (French), 天皇ダンボ (Japanese), and Kaiserdumbo (German).

#### DNA barcoding and phylogenetic analysis

**Fig. 4:**
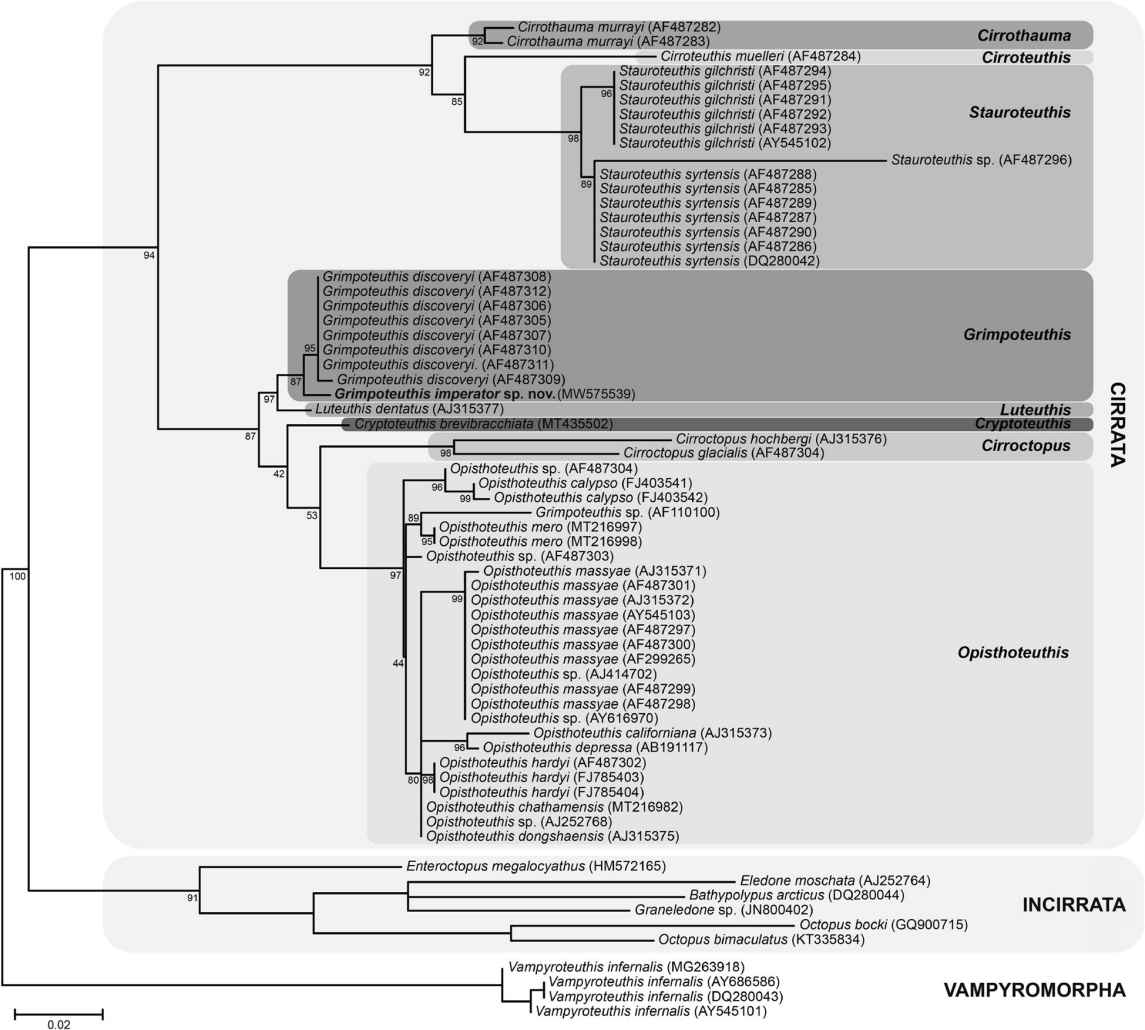
Phylogenetic analysis of the Cirrata and other selected Octopodiformes based on the 16S rRNA gene sequence. Scale bar refers to a phylogenetic distance of 0.02 nucleotide substitutions; new species marked in bold font; numbers on the branches denote bootstrap values after 500 replicates

In addition to the non-invasively acquired morphological character states, minimally invasive DNA barcoding was used to obtain the first molecular sequence data for an unambiguously identified member of the genus *Grimpoteuthis* from the Pacific Ocean. Inferences based on this as well as 55 previously deposited 16S rRNA gene sequences result in the first phylogeny with full coverage of all eight extant cirrate genera (Fig. 4).

The phylogenetic analysis provided very good resolution within and between cirrate genera as well as all outgroup taxa. The North Atlantic *Grimpoteuthis* specimens are all placed in a well-supported clade (95% bootstrap value). The new species *G. imperator* sp. nov. from the North Pacific is sister to this North Atlantic *Grimpoteuthis* clade (87%). Sister to all *Grimpoteuthis* species is *Luteuthis dentatus*, a branching that is statistically well-supported (97%). This larger clade in turn is sister to several species of the genera *Cryptoteuthis*, *Cirroctopus*, and *Opisthoteuthis*, a branching supported by a high bootstrap value as well (87%). A further, well supported (92%) clade composed of species from the cirrate genera *Cirrothauma*, *Cirroteuthis*, and *Stauroteuthis* is then sister to the clade composed of all previously mentioned cirrate taxa. Furthermore, all incirrate species included in the present analysis are sister to the well supported cirrate clade (94%). Finally, the vampire squid *Vampyroteuthis* is sister to all octopod species (100%).

Interspecific uncorrected p-distances based on the 16S rRNA gene fragment between *G. imperator* sp. nov. and *Grimpoteuthis* specimens from the North Atlantic vary between 2.2 and 2.5%. Interspecific distances between *G. imperator* sp. nov. and *L. dentatus* are 3.2% and between *G. imperator* sp. nov. and other cirrate species range from 8.9% (*Opisthoteuthis depressa*) to 16.6% (*Stauroteuthis syrtensis*). For reference, interspecific distances within other genera lie between 0.9% (*S. syrtensis* and *S. gilchristi*) and 4.3% (*O. hardyi* and *O. californiana*).

## Discussion

In recent years, several non-invasive imaging techniques have become available [4–8] that permit acquisition of high quality structural data in 3D from various metazoan taxa, including terrestrial and aquatic megafauna [33, 35–37]. In the present study, MRI was performed overnight using a high-field clinical imaging system [33]. Potentially harmful long-term effects on the singular specimen were avoided by scanning the organism in its original fixative without any contrast agent added. At 140 μm isotropic voxel resolution, the signal to noise ratio of the resulting 3D dataset was excellent (Fig. 1f), allowing extraction of all taxonomically relevant internal morphological characters such as shell and gill shape or digestive tract morphology, but also of more minute structures such as nervous system and sensory organ morphology (Fig. 2c). In addition, morphological characters so far not used in cirrate species descriptions, but potentially of taxonomic value such as the shape of the systemic heart or the arrangement of the dorsal aorta [38], were here integrated into the description of a new cirrate species for the first time as well (Fig. 1k).

The capacity of MRI to visualize water-rich tissues has in the past been applied to a broad spectrum of zoological specimens using different MR modalities such as post mortem, in vivo, or diffusion tensor imaging [4–8, 15, 20, 33, 35–37, 39–41]. However, due to the tissue properties of cephalopod beaks and radula (i.e. water-poor chitin) as well as a strong susceptibility artefact in the buccal mass area (Fig. 1f) caused by ingested sediment [36, 41], a complementary region of interest μCT scan was conducted at 10.93 μm isotropic voxel resolution. The improved hard part contrast of this X-ray-based imaging technique proved more suitable for the visualization of chitinous tissues (Fig. 2) and allowed building the first interactive 3D model of a cephalopod beak (Additional file 3). Although analysis of a new megafaunal species using two complementary imaging modalities with different fields of view was successfully performed here, a single scan of the entire specimen with combined soft and hard tissue contrast would obviously have been more desirable. Such studies are bound to be possible in the near future, for example, on the upcoming BM18 beamline of the European Synchrotron Radiation Facility that will permit phase-contrast tomography at nanometre spatial resolution on specimens of up to 2.5 m length [42]. In addition, recent developments based on the implementation of deep neural networks are poised to significantly increase the efficiency of semi-automated 3D dataset segmentation [43].

Apart from allowing 2D virtual sectioning at arbitrary angles (Figs. 1f, g; 2c, h, m), volume rendering of selected areas (Figs. 1i–k; 2e, n), or the creation of interactive 3D models (Additional files 2, 3), the MRI and μCT datasets underlying the present species description can be employed for further analyses that would not have been possible to this extent using conventional, invasive techniques. For example, volumetric data rapidly obtainable from the organ systems reconstructed here could be compared with similar data previously gathered non-invasively from a *Grimpoteuthis* hatchling using high-field preclinical MRI [44]. This type of analysis would permit drawing conclusions on the lifestyle and behaviour of these hard-to-observe deep-sea organisms [45].

In general, such inferences are undoubtedly facilitated by the deposition of the respective digital raw datasets in online repositories [5, 33, 46, 47], an approach of particular importance for zoological taxonomy [10–12]. Apart from allowing improved or ideally full data transparency as well as data mining and modelling, dataset deposition in online repositories could in the future permit designation of so-called cybertypes [48]. Also termed virtual or e-type [49], a cybertype constitutes a digital, graphical representation of the physical specimen on which a description is based [10, 11]. Whilst the International Commission on Zoological Nomenclature presently does not consider digital copies of physical specimens as sufficiently adequate type material [50], the provision of digital cybertype infrastructure has nonetheless been identified as an important step for overcoming the so-called taxonomic impediment [51]. The two publicly accessible digital repositories for molecular (NCBI GenBank) and protein sequence data (RCSB Protein Data Bank) provide striking evidence for the benefit of a centralized and professionally curated online database [5, 48]. In the meantime, the data gathered in the course of this study were deposited on MorphoBank [52].

## Conclusions

By extending the morphomics concept to the description of a new species of megafauna, we here show that a minimally invasive approach based on the application of complementary non-invasive 3D imaging techniques supplemented with molecular sequence data can help to advance metazoan taxonomy, in particular, in cases where valuable, larger zoological specimens require a more detailed, holistic analysis.

## Supplementary Information

**Additional file 2. **Interactive 3D model of selected internal organs of *Grimpoteuthis imperator* sp. nov. (ZMB MOLL 240160). Left-click anywhere on this figure to open the embedded, interactive 3D PDF model (requires Adobe Reader 9 or higher on all operating systems). Use mouse wheel to zoom in or out. A right-click on the activated model provides access to further options such as a set of pre-saved views, a full-screen mode, the model tree icon, or deactivation of the 3D model. Branchial glands = dark green, branchial hearts = light brown, branchial heart appendages = maroon, central nervous system = medium yellow, circulatory system = dark brown, digestive gland = blue, digestive tract = dark blue, eyeballs = orange, fin cartilages = pink, gills = light green, hepatic ducts = turquoise, lenses = dark grey, optic lobes = dark yellow, pancreas = light blue, peripheral nervous system = light yellow, posterior salivary gland = bluegreen, renal appendages = magenta, reproductive system = light grey, shell = red, statocysts = purple, ureters = violet, white bodies = white.

**Additional file 3. **Interactive 3D model of upper and lower beak of *Grimpoteuthis imperator* sp. nov. (ZMB MOLL 240160). Left-click anywhere on this figure to open the embedded, interactive 3D PDF model (requires Adobe Reader 9 or higher on all operating systems). Use mouse wheel to zoom in or out. A right-click on the activated model provides access to further options such as a set of pre-saved views, a full-screen mode, the model tree icon, or deactivation of the 3D model.

## Data Availability

Additional imagery of the specimen as well as the MRI and μCT 8-bit TIFF image stacks are available for download at the MorphoBank project account accompanying this publication [32]. Please select the ‘Media’ and ‘Documents’ tabs to access the photographs and image stacks as well as Adobe PRW and Amira AM label field files, respectively. Furthermore, all gene sequence data gathered in the course of this study were uploaded to GenBank under accession numbers MW575539 (16S rRNA) and MT570977 (COI). In addition, the new species (LSID urn:lsid:zoobank.org:act:6A09F5C2-314C-4854-BAEC-E3BDD7E5993E) as well as the present publication (LSID urn:lsid:zoobank.org:pub: E8179739-FD5B-4135-96D5-DB6B41D11233) were registered on ZooBank.
