# Supplementary figures and images for: Holistic description of new deep sea megafauna (Cephalopoda: Cirrata) using a minimally invasive approach
Source: BMC Biol. 2021 Apr 23;19:81. doi: 10.1186/s12915-021-01000-9 (PMC8063452; doi:10.1186/s12915-021-01000-9)

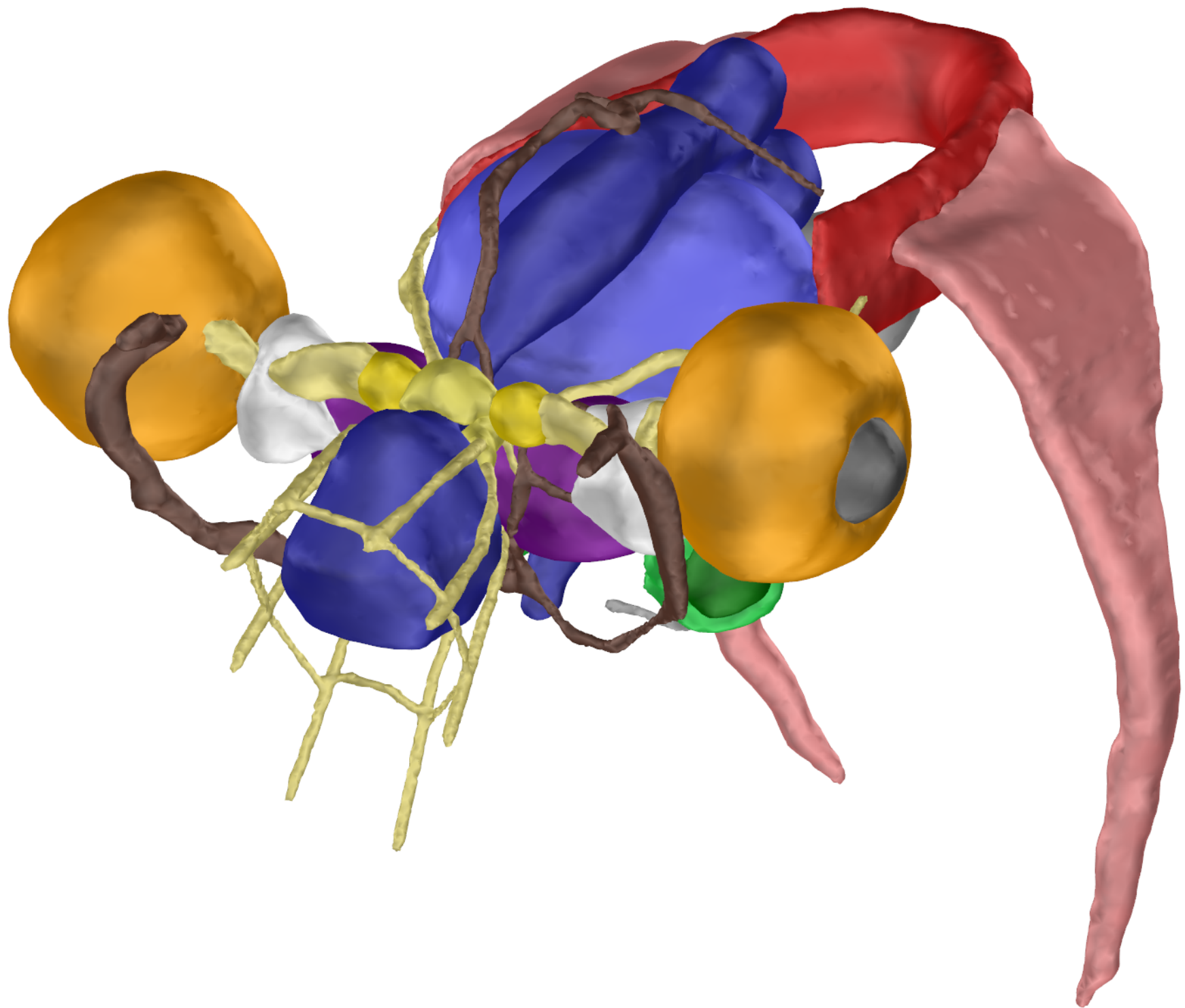

Supplement: Supplementary file 2 — Additional file 2. Interactive 3D model of selected internal organs of Grimpoteuthis imperator sp. nov. (ZMB MOLL 240160). Left-click anywhere on this figure to open the embedded, interactive 3D PDF model (requires Adobe Reader 9 or higher on all operating systems). Use mouse wheel to zoom in or out. A right-click on the activated model provides access to further options such as a set of pre-saved views, a full-screen mode, the model tree icon, or deactivation of the 3D model. Branchial glands = dark green, branchial hearts = light brown, branchial heart appendages = maroon, central nervous system = medium yellow, circulatory system = dark brown, digestive gland = blue, digestive tract = dark blue, eyeballs = orange, fin cartilages = pink, gills = light green, hepatic ducts = turquoise, lenses = dark grey, optic lobes = dark yellow, pancreas = light blue, peripheral nervous system = light yellow, posterior salivary gland = bluegreen, renal appendages = magenta, reproductive system = light grey, shell = red, statocysts = purple, ureters = violet, white bodies = white. [file 12915_2021_1000_MOESM2_ESM.pdf]

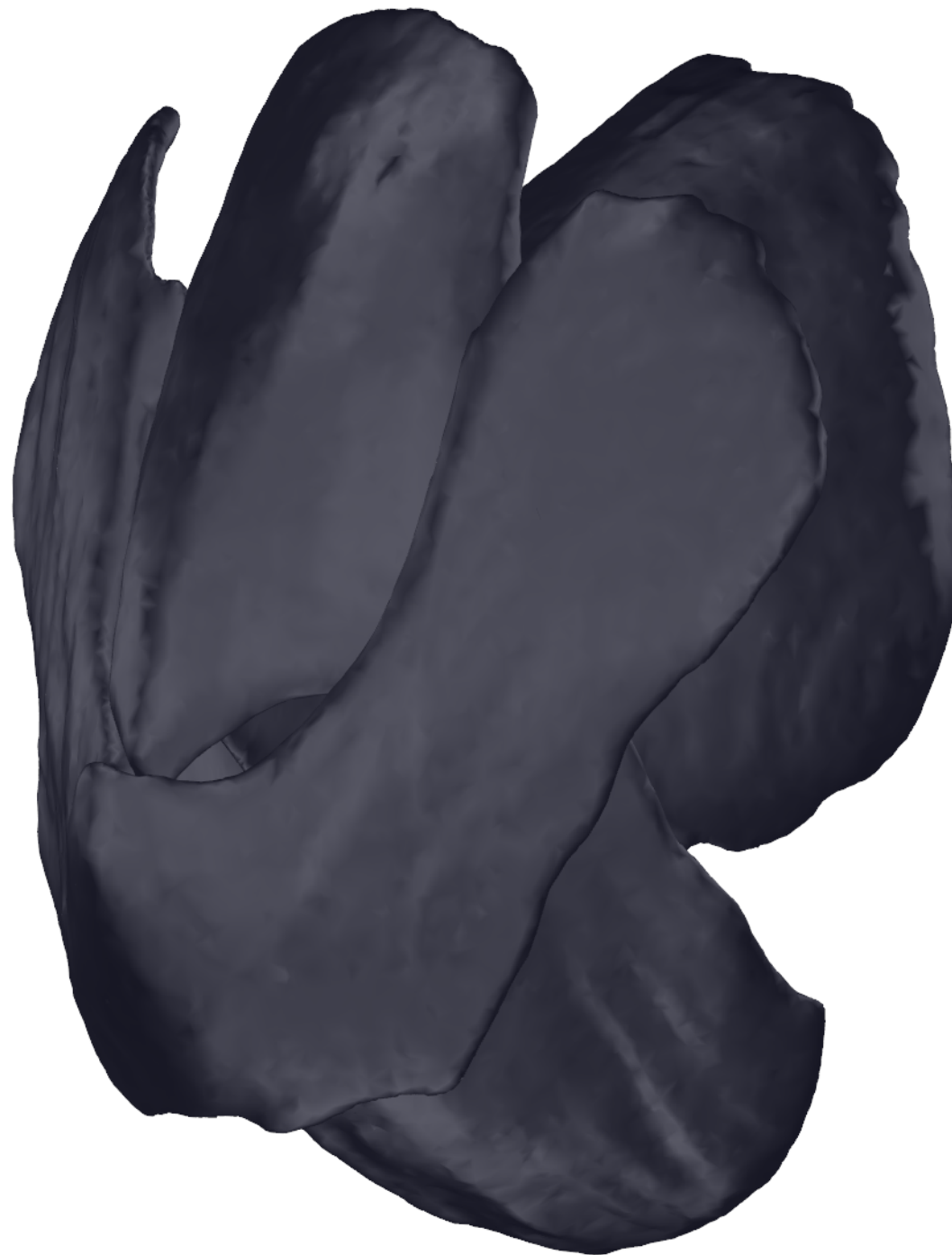

Supplement: Supplementary file 3 — Additional file 3. Interactive 3D model of upper and lower beak of Grimpoteuthis imperator sp. nov. (ZMB MOLL 240160). Left-click anywhere on this figure to open the embedded, interactive 3D PDF model (requires Adobe Reader 9 or higher on all operating systems). Use mouse wheel to zoom in or out. A right-click on the activated model provides access to further options such as a set of pre-saved views, a full-screen mode, the model tree icon, or deactivation of the 3D model. [file 12915_2021_1000_MOESM3_ESM.pdf]
